# Socioeconomic indicators and economic investments influence oral cancer mortality in Latin America

**DOI:** 10.1186/s12889-021-10419-2

**Published:** 2021-02-18

**Authors:** Aldelany Ramalho Freire, Deborah Ellen Wanderley Gomes Freire, Elza Cristina Farias de Araújo, Fernanda Campos de Almeida Carrer, Gilberto Alfredo PuccaJúnior, Simone Alves de Sousa, Edson Hilan Gomes de Lucena, Yuri Wanderley Cavalcanti

**Affiliations:** 1 Clinicaland Social DentistryDepartment, DCOS/CCS/UFPB, Federal University of Paraíba, Cidade Universitária, Campus I, João Pessoa, PB 58051-900 Brazil; 2 Social Dentistry Department, University of São Paulo, Av. Prof. Lineu Prestes - FOUSP, São Paulo, SP 05508-000 Brazil; 3 Department of Dentistry, UnB, Brasilia University, Brasília, DF 70297-400 Brazil

**Keywords:** Oral cancer, Mortality, Latin America, Socioeconomic factors

## Abstract

**Background:**

It is necessary to recognize the influence of socioeconomic factors on oral cancer indicators in Latin American countries. This study aimed to analyze the influence of socioeconomic indicators and economical investments on oral cancer mortality rates in Latin American countries.

**Methods:**

This cross-sectional study considered the age-standardized mortality rate (ASR) of oral cancer within the period 2000–2015. The oral cancer mortality rate (for both sexes and age groups 40–59 and 60 years old or more), socioeconomic aspects (Gini Inequality Index, unemployment rate and Gross Domestic Product (GDP) per capita) and investments in different sectors (%GDP invested in health per capita and by the government, %GDP invested in education by the government and %GDP invested in research and development) were considered. Tweedie multivariate regression was used to estimate the effect of independent variables on the mortality rate of oral cancer, considering *p* < 0.05.

**Results:**

This study showed that being male and aged 60 or over (PR = 14.7) was associated with higher mortality rate for oral cancer. In addition, greater inequality (PR = 1.05), higher health expenditure per capita (PR =1.09) and greater investment in research and development (PR = 1.81) were associated with a higher mortality rate from oral cancer.

**Conclusion:**

Socioeconomic factors and economical investments influence the mortality rate of oral cancer in Latin American countries. This emphasizes oral cancer is a socioeconomic-mediated disease.

## Background

Oral cancer has high rates of morbidity and mortality around the world, and is therefore considered a growing public health problem [1, 2]. The disease’s treatment often has a significant impact on the quality of life of individuals, which is affected by mutilations, functional limitations and chronic pain [3].

Oral cancer has a complex and multifactorial etiology, with tobacco and alcohol consumption being the most relevant factor [4, 5]. In recent decades, the discussion on the role of socioeconomic factors as determinants for the disease has intensified, suggesting that socioeconomically vulnerable individuals are at increased risk for oral cancer and its complications [6–8]. The greater exposure to risk factors, limited access to health services and consequent low frequency of early diagnosis might explain the relationship between socioeconomic factors and incidence of oral cancer [9, 10].

Latin America has shown recent advances in the health conditions of its population, with a reduction in infant mortality and an increase in life expectancy [11]. However, strong social inequalities and segmentation in their health systems still persist, in which a large part of the population (non-wage earners) does not have full access to health services [12, 13]. In addition, the organization of health services and government investments differ between countries, contributing to great inequity in health in the region [12–14].

In this scenario, Latin America is characterized as a region with a high incidence of oral and oropharynx cancer [15]. Between 2018 and 2025, a 22.4% increase in the number of new cases of the disease in the region is expected. As for mortality, the expected growth is 24.1% for the same period [16]. Cuba, Brazil, Puerto Rico and Uruguay occupy, in that order, the first positions regarding the highest age-standardized incidence rates (ASR) [16]. Cuba and Brazil also lead the most recent estimates regarding mortality by the disease in the region [16].

Therefore, it is necessary to recognize the influence of socioeconomic factors on oral cancer indicators in Latin American countries. Thus, policies to combat social inequalities and public health measures for prevention, early diagnosis and treatment can be targeted, seeking to reduce cancer rates in the population [17]. However, it appears that several Latin American countries have adopted austerity policies, which limit investments and public spending. This situation can result in an increase in inequality and worsening in the epidemiological panorama of the population [18, 19].

Thus, the aim of this study was to analyze the influence of socioeconomic indicators and government investments on oral cancer mortality rates in Latin American countries.

## Methods

### Study design

An observational cross-sectional retrospective study was carried out, considering the time period between the years 2000 and 2015 (totaling 16 years analyzed). The sample units of this study were the countries located in Latin America and the Caribbean, whose data regarding mortality from oral cancer, socioeconomic aspects and government investments were available in international databases.

### Data collection

The data referring to the number of deaths from oral cancer (considering the category lip, oral cavity and pharynx) of each country, for both sexes and the age groups of 40–59 years and 60 years or more, were collected from the World Health Organization Cancer Mortality Database (*http://www-dep.iarc.fr/WHOdb/WHOdb.htm*), considering each year of the period between 2000 and 2015. The Gini inequality coefficient, unemployment rate, Gross Domestic Product (GDP) per capita, GDP percentage invested in health per capita, GDP percentage invested in health by the government, GDP percentage invested in education by the government and GDP percentage invested in research and development were extracted from the World Bank indicator system (*https://data.worldbank.org/indicator*).

### Constitution of the model

The response variable (dependent) of this study was the oral cancer mortality rate, expressed through the standardized mortality rate. The independent variables were: sex, age, period (year), Gini inequality coefficient, unemployment rate, Gross Domestic Product (GDP) per capita, GDP percentage invested in health per capita, GDP percentage invested in health by the government, GDP percentage invested in education by the government and GDP percentage invested in research and development.

### Statistical analysis

The data were put in charts and analyzed using the IBM Statistical Package for Social Sciences program (IBM SPSS, v. 24, IBM, Chicago, IL). Tweedie’s Multivariate Regression was used to estimate the effect of independent variables on the mortality rate among Latin American countries. The model was adjusted by gradually removing variables with a *p*-value> 0.20. The effect of the independent variables was verified through the measures of prevalence ratio (PR) and confidence interval (CI95%), considering p-value< 0.05.

## Results

Descriptive data regarding the oral cancer standardized mortality rate for each Country is presented in Table 1. Descriptive data regarding the socioeconomic parameters of Latin American countries considered for this study is presented in Table 2. Factors associated to the standardized mortality rate of oral cancer (mouth, lip and oropharynx) are presented in Table 3. The adjusted model did not account for the effect of year and unemployment rate, since those variables presented p-value> 0.20 during model adjustment. Gross Domestic Product per capita, Government Health Expenditure (as %GDP) and Government Education Investments (as %GDP) did not influence the oral cancer mortality rate within the adjusted model. Male population with 60 years or more is associated with higher mortality of oral cancer. Greater inequality (greater Gini index), higher health expenditure per capita (as %GDP) and higher investments on research and development (%GDP) are associated with higher oral cancer mortality rate among Latin American Countries.

**Table 1:** Standardized mortality rate and tendency of oral cancer in Latin American Countries, considering the period between 2000 and 2015, according to populations’ sex and age

	Female				Male			
	40 to 59 years		60 years and above		40 to 59 years		60 years and above	
	Mean (SD)	Tendency	Mean (SD)	Tendency	Mean (SD)	Tendency	Mean (SD)	Tendency
Argentina	1.02 (0.13)	=	4.38 (0.33)	=	5.36 (1.02)	↓	16.72 (1.47)	↓
Brazil	1.58 (0.12)	=	7.25 (0.47)	↓	11.50 (0.44)	↓	29.80 (1.16)	=
Chile	0.56 (0.18)	↑	3.14 (0.42)	=	1.99 (0.42)	↓	11.28 (1.23)	↓
Colombia	0.86 (0.21)	=	6.38 (0.92)	↓	1.87 (0.23)	=	11.42 (1.36)	↓
Ecuador	0.59 (0.17)	=	3.86 (0.93)	=	0.99 (0.25)	=	5.88 (1.30)	=
Guatemala	1.04 (0.44)	=	5.35 (1.67)	=	1.82 (0.87)	↓	10.78 (2.57)	↓
Mexico	0.73 (0.09)	↓	3.87 (0.39)	=	1.78 (0.17)	=	9.54 (0.94)	↓
Nicaragua	0.52 (0.35)	=	2.54 (1.01)	↑	2.01 (0.80)	=	8.50 (2.11)	=
Panama	0.81 (0.70)	=	6.03 (1.74)	↓	2.40 (1.19)	↑	15.17 (2.91)	=
Paraguay	0.55 (0.39)	=	3.58 (1.32)	=	4.34 (0.86)	=	17.11 (3.44)	=
Peru	0.82 (0.24)	↑	3.60 (1.15)	↑	0.90 (0.28)	↑	5.40 (0.77)	=

Mean (SD): Mean and Standard Deviation of Standardized mortality rate. ↑: Increasing tendency. =: Stable tendency. ↓: Decreasing tendency. Tendency was defined according to the World Health Organization Cancer Mortality Database (http://www-dep.iarc.fr/WHOdb/WHOdb.htm)

**Table 2:** Descriptive data regarding the socioeconomic parameters of Latin American countries considered for this study, considering the period between 2000 and 2015

	Gross Domestic Product (GDP) *per capta*		Gini Index		Unemployment rate		Health expenditure *per capta*(%GDP)		Government Health Expenditure (as %GDP)		Government Education Investments (as % GDP)		Investments on Research and Development (as %GDP)	
	Mean	SD	Mean	SD	Mean	SD	Mean	SD	Mean	SD	Mean	SD	Mean	SD
Argentina	8408.903	3259.514	46.78	4.13	11.504	4.007	8.071	0.588	5.020	1.002	4.72	0.72	0.487	0.084
Brazil	7052.029	3541.317	55.87	2.87	8.467	1.124	8.805	1.360	3.301	0.631	4.97	0.87	1.077	0.104
Chile	9098.673	4085.495	50.69	2.95	8.660	1.631	7.035	0.548	3.946	0.425	3.94	0.51	0.331	0.029
Colombia	4466.826	2164.274	54.89	2.07	12.639	3.291	5.759	0.307	4.119	0.259	4.26	0.40	0.204	0.061
Ecuador	3573.372	1599.684	51.12	4.20	4.221	0.764	6.319	1.693	2.617	1.199	2.74	1.83	0.195	0.152
Guatemala	2382.026	778.592	53.66	2.05	2.970	0.334	6.153	0.309	2.083	0.095	2.27	0.72	0.041	0.010
Mexico	8208.664	1873.468	47.36	2.13	4.012	0.937	5.530	0.402	2.621	0.338	4.83	0.36	0.410	0.091
Nicaragua	1312.002	363.456	48.99	4.07	6.491	1.216	6.340	1.010	3.128	0.764	3.22	0.92	0.073	0.024
Panama	6703.067	3242.234	53.98	2.50	3.664	0.736	6.740	0.264	4.393	0.293	3.62	0.46	0.216	0.100
Paraguay	3106.709	1674.421	52.23	2.77	6.580	2.230	6.012	1.025	2.742	0.848	4.14	0.64	0.077	0.018
Peru	3707.913	1794.793	49.13	4.15	4.479	1.016	4.803	0.252	2.600	0.320	3.03	0.36	0.106	0.029

*GDP* Gross Domestic Product, *SD* Standard Deviation

**Table 3:** Factors associated to the oral cancer (mouth, lip and oropharynx) standardized mortality rate in Latin American countries^a^, between 2000 and 2015. Tweedie multiple regression analysis was used to estimate the effect of socioeconomic factors and economic investments on the mortality of oral cancer

	Unadjusted Model (all variables)					Adjusted Model (variables with *p* < 0.20)				
Parameter	B	*p*-value	PR	95% CI		B	*p*-value	PR	95% CI	
				Lower	Upper				Lower	Upper
Male, 60 years or more	2.690	< 0.001	14.737	13.550	16.028	2.691	< 0.001	14.747	13.560	16.037
Female, 60 years or more	1.681	< 0.001	5.370	4.912	5.871	1.681	< 0.001	5.370	4.914	5.869
Male, 40 to 59 years	1.237	< 0.001	3.446	3.095	3.836	1.238	< 0.001	3.449	3.098	3.839
Female, 40 to 59 years			Ref.					Ref.		
Year (2000 to 2015)	−0.004	0.337	0.996	0.988	1.004					
Gross Domestic Product (GDP) *per capta*	−6.385E-6	0.303	1.000	1.000	1.000	−8.587E-6	0.132	1.000	1.000	1.000
Gini Index	0.046	< 0.001	1.047	1.037	1.056	0.047	< 0.001	1.048	1.039	1.057
Unemployment rate	0.000	0.967	1.000	0.990	1.010					
Health expenditure *per capta*(%GDP)	0.088	< 0.001	1.092	1.048	1.138	0.082	< 0.001	1.085	1.044	1.129
Government Health Expenditure (as %GDP)	0.027	0.264	1.027	0.980	1.077	0.032	0.126	1.032	0.991	1.075
Government Education Investments (as % GDP)	0.048	0.044	1.050	1.001	1.100	0.045	0.063	1.046	0.998	1.096
Investments on Research and Development (as %GDP)	0.567	< 0.001	1.763	1.439	2.160	0.594	< 0.001	1.811	1.496	2.192

Statistical significance of the adjusted model was set at 5%. ^a^Latin American countries: Argentina, Brazil, Chile, Colombia, Ecuador, Guatemala, Mexico, Nicaragua, Panama, Paraguay and Peru. *GDP* Gross Domestic Product, *B* Regression Coeficient, *PR* Prevalence ratio, *95% CI* 95% Confidence interval

## Discussion

The results of this investigation demonstrate that socioeconomic factors and government investments influence mortality by oral cancer in Latin American countries. The results of this study reaffirm the higher prevalence of oral cancer among male individuals, over 60 years old [15, 20–22]. Also, it revealed that social inequalities are related with the increase of oral cancer mortality in these countries. Although government investments in health and education have not influenced standardized mortality rates, investments per capita in health and spending on research and development seem to be the result of the expansion of austerity policies and less government accountability for the population’s health problems.

The choice of the evaluation period for this study is justified by the fact that the years 2000 to 2015 corresponded to the most recent time interval in which the largest number of Latin American countries presented data available on the analyzed databases. During this period, mortality by oral cancer among the countries included was stable. Previous studies have shown great variability in the trend of mortality from oral cancer in Latin America in the last decades [15, 23, 24]. This is probably due to the absence or discontinuity of records in some countries, in addition to the singularities of each country, especially regarding the distribution of risk factors in the region.

In this study, the Gross Domestic Product (GDP) showed no association with mortality from oral cancer, suggesting that this condition is not influenced by the amount of wealth in the countries. The comparison between Cuba and Brazil could illustrate this finding: despite the great difference in the economic organization and wealth of these countries, both lead the indicators of mortality from oral cancer in Latin America. However, it is important to consider that Latin countries are marked by strong socioeconomic inequalities, which was evidenced in this study through the Gini Index, which was positively associated with mortality.

Thus, although there is no difference between poor and rich countries, inequality is an important factor. The same is true for the unemployment rate, which, despite not being part of the association model, is an important indicator of inequality and may have been included in the Gini Index. A similar study found no association between this index and mortality from oral cancer in Latin America [10]. However, the authors of the aforementioned study suggested that a regional analysis may not be able to detect this association, making it necessary to conduct national studies to better explain this relationship.

Government investments in education and health have not influenced the mortality rate and may suggest that in these countries investments in social policies and health systems are still low and insufficient, unable to meet the demands of the population and contribute to the improvement of their health condition [25]. A positive association was verified between oral cancer mortality and health expenditure per capita, which means that the countries that spent more on health also had higher mortality. This finding suggests that health care in the private sector has higher cost for population and not adequate efficiency [11].

Another intriguing finding refers to the positive association between investment in research and development and mortality from oral cancer. This association was probably influenced by the fact that Brazil is the country with the highest investment in research and development, also presenting the highest mortality rate from oral cancer among the countries analyzed. Although there is investment in the sector, it does not seem to positively influence the population’s health, and it is necessary to analyze this finding individually for each country, along with its other characteristics. It is also important to reflect on the use of strategies so that the products of scientific health development in these countries become accessible to the majority of the population, thus providing a real impact on the indicators of cancer and other diseases [26].

Despite the recognition of socioeconomic conditions as determining factors for oral cancer, the presence of harmful habits related to lifestyle are the strongest etiological factors [4, 27]. Latin countries in which public policies for the control of tobacco and alcohol consumption have been developed have shown less advances in mortality rates from oral cancer in the last decades [14]. Therefore, socioeconomic factors must always be analyzed in the light of the population’s habits that are involved. In addition, policies to control risk factors must be implemented in conjunction with policies to combat social inequalities. Based on that, campaigns to fight tobacco use, limit sun exposition, reduce alcohol ingestion, and extend access to healthcare should be outlined simultaneously with the reduction of social inequalities.

This study presents the proper limitations of a cross-sectional study based on data from an information system, being liable to failures due to the secondary origin of the data. However, databases and information systems considered world reference were used, seeking to ensure greater reliability. The analysis of the Latin American region also requires care in interpreting the results, since not all countries were included due to the lack of data, and the particularities of each country must be considered. As previously discussed, the presence of habits that represent risk factors for oral cancer should be studied in conjunction with socioeconomic determinants. Therefore, it is suggested to conduct further studies that consider both aspects.

The analysis of oral cancer mortality in Latin America from the perspective developed in this study proposes important reflections on the austerity policies in public health investments that are being implemented in the region. Previous experiences in European countries have demonstrated the damage these policies have to the living and health conditions of the population, worsening socioeconomic inequities, intensifying unemployment, violence, social unprotection, decreasing access and coverage of health services, among others [28, 29]. Neoliberal measures have threatened the main universal health system in the world, Brazil’s Unified Health System [19]. The scientific debate on the new health challenges in Latin America is necessary, aiming at the preservation of achievements resulting from past social struggles, such as social protection, strengthening of health systems, improvement in health indicators and achieving international development goals [28].

In conclusion, this manuscript highlights socioeconomic factors and economical investments influence the mortality rate of oral cancer in Latin American countries. This emphasizes oral cancer is a socioeconomic-mediated disease. Austerity policies may play a significant role on the mortality rate of oral cancer in Latin America.

## Data Availability

The datasets generated during the current study can be provided by the corresponding author under reasonable request. Data were extracted from the following websites: https://www-dep.iarc.fr/WHOdb/WHOdb.htm and https://data.worldbank.org/indicator.

## Abbreviations

95% C.I.: 95% Confidence Interval
ASR: Age-Standardized Incidence Rates
B: Regression Coefficient
GDP: Gross Domestic Product
PR: Prevalence Ratio
SD: Standard Deviation
WHO: World Health Organization
